# Supplementary material for: Functional symmetrization of neuromotor modules during locomotor development in human infants
Source: Commun Biol. 2025 Dec 18;8:1782. doi: 10.1038/s42003-025-09198-y (PMC12714737; doi:10.1038/s42003-025-09198-y)
Supplement: Supplementary file 2 — Supplementary Information [file 42003_2025_9198_MOESM2_ESM.pdf]

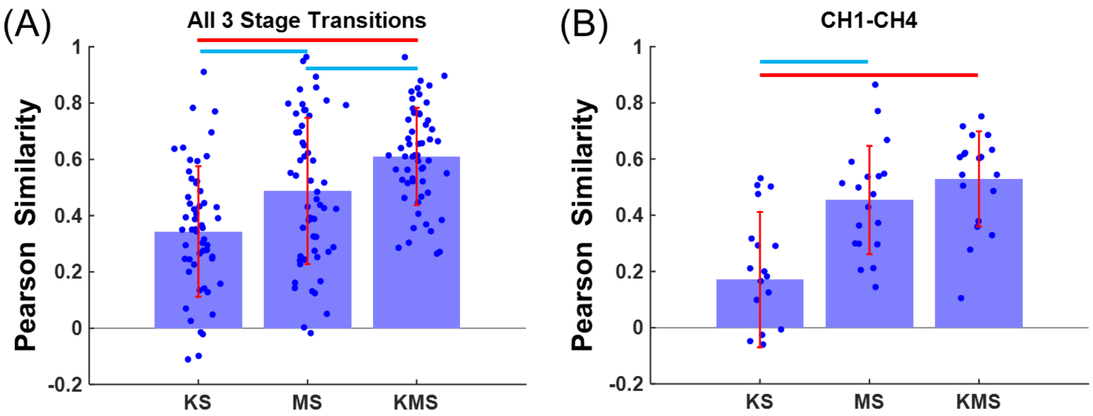

Fig. S1. Between-Stage Synergy Similarity. (A) To assess developmental changes in synergy patterns, we evaluated the between-stage similarity, quantified by the Pearson correlation, for KS, MS, KMS across all three stage transitions (Fig. 2C). **(B)** Changes in KS, MS, and KMS from CH1 to CH4 were quantified by calculating the Pearson correlation between synergies at CH1 and CH4. Plot formats are consistent with those in Fig. 2C.

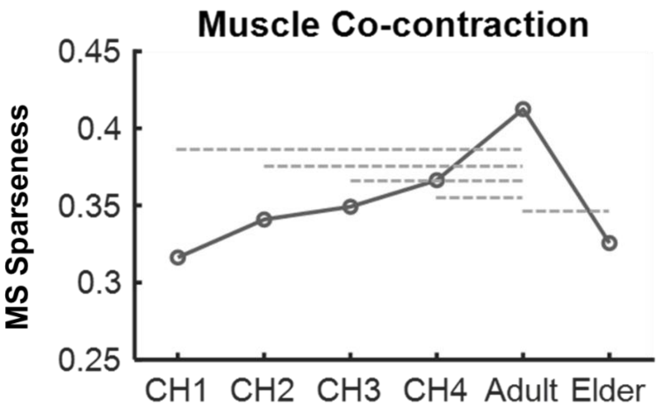

Fig. S2. Sparseness of MS Across the Lifespan. We quantified the sparseness of each MS to assess the degree of independent muscle control, with higher sparseness indicating more selective activation of specific muscles. MS sparseness was compared across the six life stages to evaluate age-related changes in muscle co-contraction during gait control. Adults demonstrated significantly higher MS sparseness than infants, reflecting more independent muscle control. Significant differences between stages are indicated by horizontal dotted lines ($p < 0.05$).

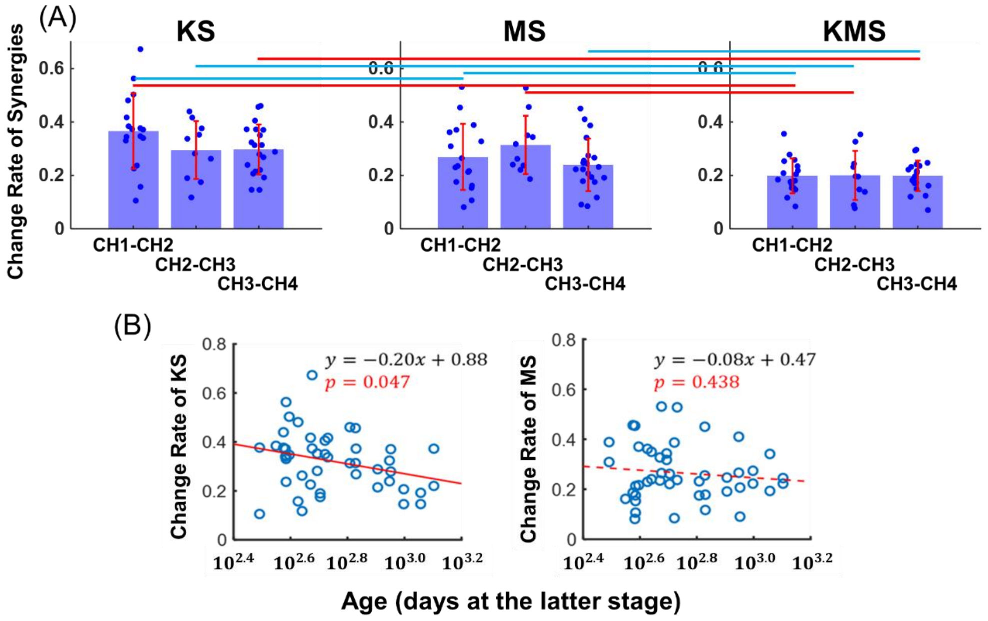

Fig. S3. Rate of Synergy Change Between Stages. To quantify the dynamics of synergy development, we calculated the rate of change for each synergy by dividing the between-stage synergy difference by the time interval between stages. **(A)** Populational means of change rates of the synergies between stages. **(B)** To assess age-related trends, we regressed the rate of synergy change against the logarithm (base 10) of the exact age at the later stage. Format matches Fig. 2C.

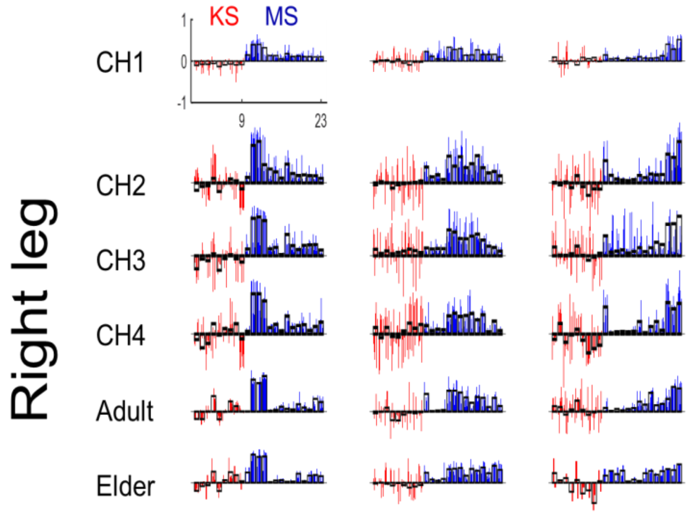

Fig. S4. *K*-means Clustering of KMSs From the Right Leg. *K*-means clustering was performed on KMSs extracted from subjects' right leg at each stage based on MS similarity. This figure parallels analogous analytic results shown for the left leg (Fig. 4A). Three clusters were identified at each stage (cluster labels shown on the left). Within each cluster, KS are displayed in red, MS in blue, and cluster centroids are represented by transparent bars.

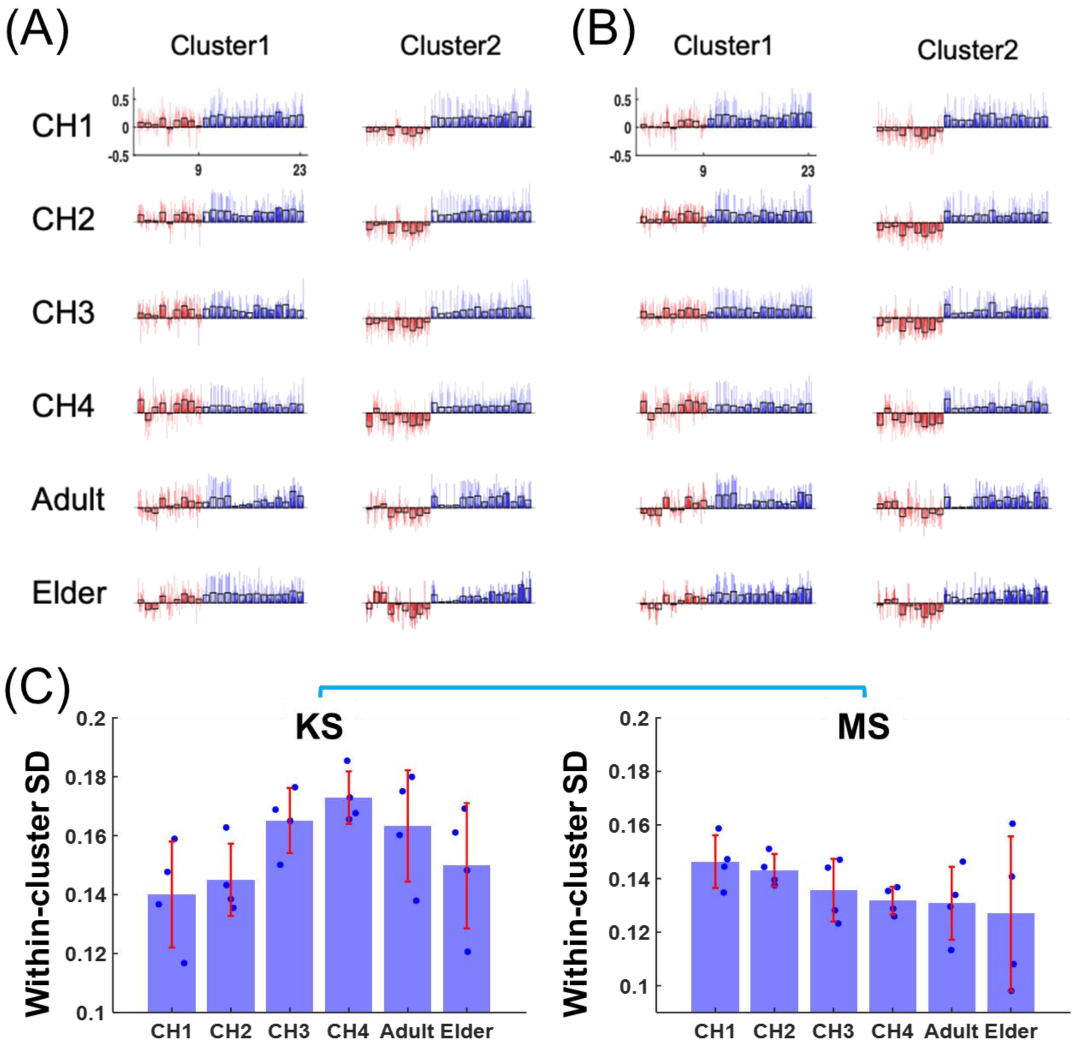

Fig. S5. *K*-means Clustering on KSs. To confirm that the greater variability observed in KSs (as compared with MS variability) in Fig. 4B was not simply due to clustering being performed on MSs, we applied *k*-means clustering directly to group similar KSs. **(A-B)** Two clusters were identified for subject cohorts at each stage for the left and right lower limbs, respectively. Due to the variable associations between KS and MS, clustering by KSs led to substantial variability in the associated MSs, resulting in cancellations from the larger and smaller MS components that produced similar averaged MSs profiles across clusters. **(C)** Although no significant changes in the within-cluster subject variability were observed across the six stages for either KS or MS, KSs still exhibited significantly greater variability than MSs even when clustering was based on KSs ($p = 0.0028$). Figure format is consistent with Fig. 4 and Fig. S4.

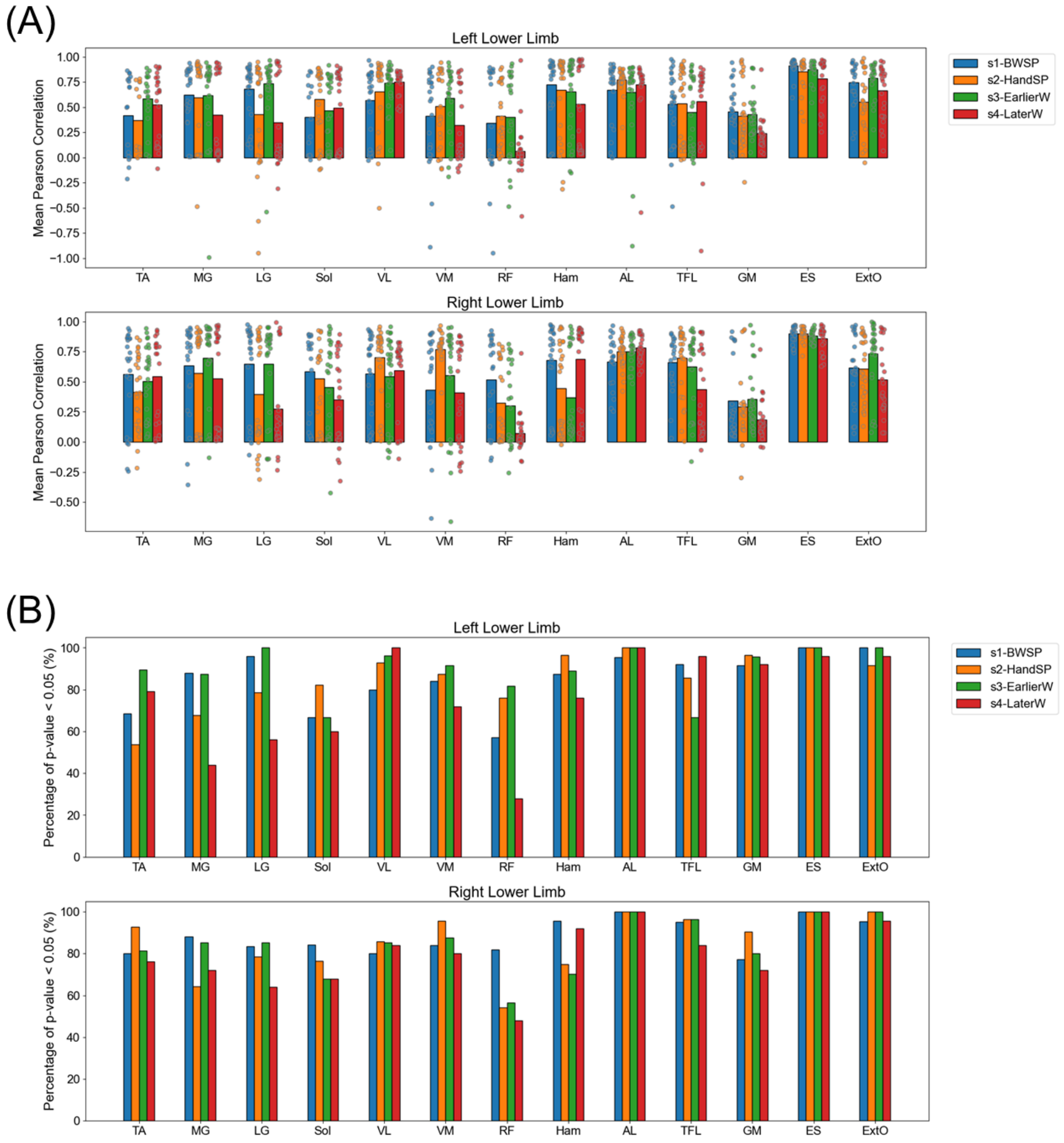

Fig. S6. Evaluating the Accuracy of NMS Model. (A) For each infant at each stage, Pearson correlation coefficients were computed between muscle activations estimated by the calibrated NMS model and experimental EMG signals, following temporal alignment using cross-correlation. The upper panel displays results for muscles of the subjects' left lower limb, and the lower panel for the right lower limb. For each muscle at each stage, the mean Pearson correlation across 11 infants and multiple validation trials is represented by a colored bar (color labels shown in the upper right). Individual data points from each validation trial of each subject are overlaid as scatter points. **(B)** For each stage and each muscle, we reported the percentage of validation trials whose correlations between model-predicted muscle activations and experimental EMGs were associated with p values of less than 0.05.

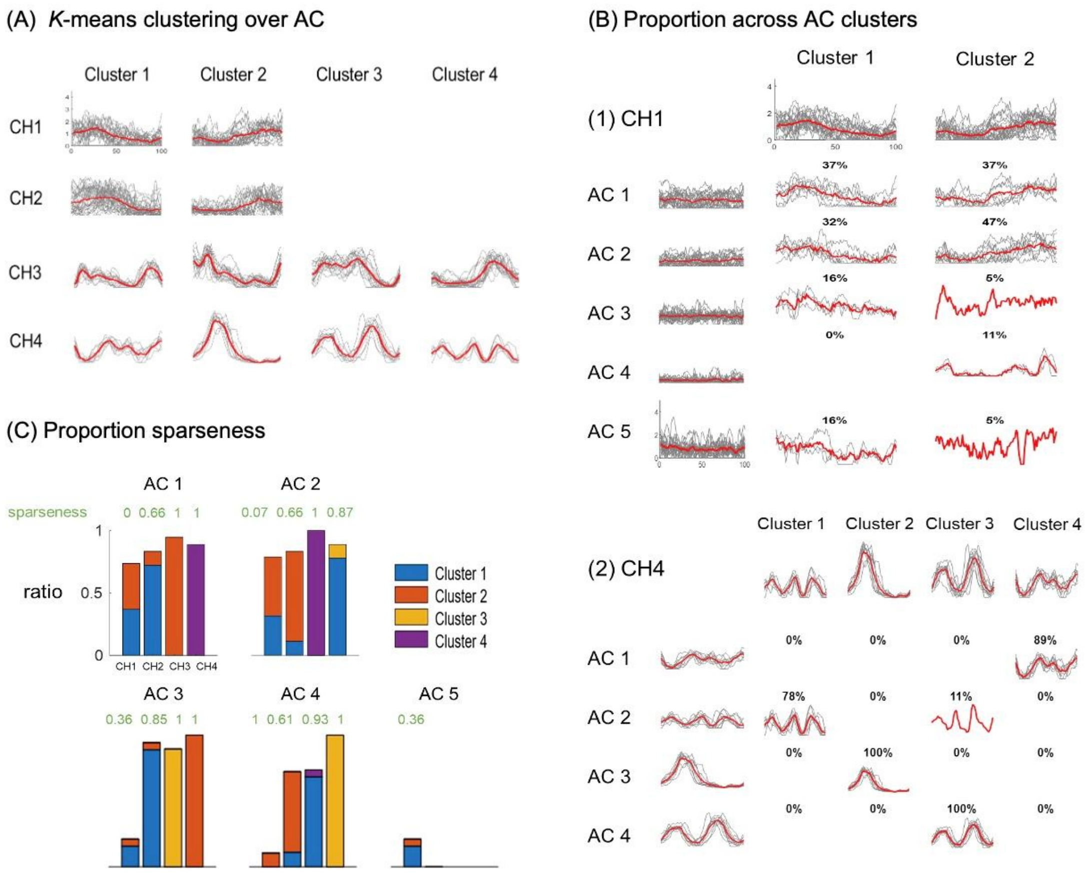

Fig. S7. Convergence of AC Patterns Across Infant Locomotor Development. (A) For each infant and developmental stage, *k*-means clustering was applied to multiple cycles of AC from all KMSs to determine the number of distinct AC patterns. As an example, four stages are shown for one subject (labels on the left): two clusters were required for CH1 and CH2, and four clusters for CH3 and CH4. Within each cluster, individual AC cycles are shown in gray, with the cluster mean in red. Statistical changes in the number of clusters throughout development are shown in Fig. 8A. **(B)** Example data for CH1 (1) and CH4 (2): For CH1 there were 5 KMSs, and for CH4, 4 KMSs. For both subplots, multiple AC cycles for each KMS are plotted on the left, with AC clusters at each stage indicated at the top. For each KMS, the proportion of AC cycles assigned to each cluster is shown at the intersection, with individual cycles and the mean profile presented as in **(A)**. **(C)** Each subplot displays the distribution of AC cycles across clusters for each KMS, with one bar representing each infantile stage. Proportions across four clusters are color-coded (Cluster 1/2/3/4: blue/red/yellow/purple). The sparseness of this distribution (shown above each bar in green) was calculated for each KMS at each stage, reflecting the concentration of AC cycles within a single cluster and thus the stability of temporal patterns. Statistical analysis across all subjects revealed that sparseness increased with age, indicating that temporal modulation of KMS becomes more consistent during gait development (see Fig. 8B, Pattern Stability).

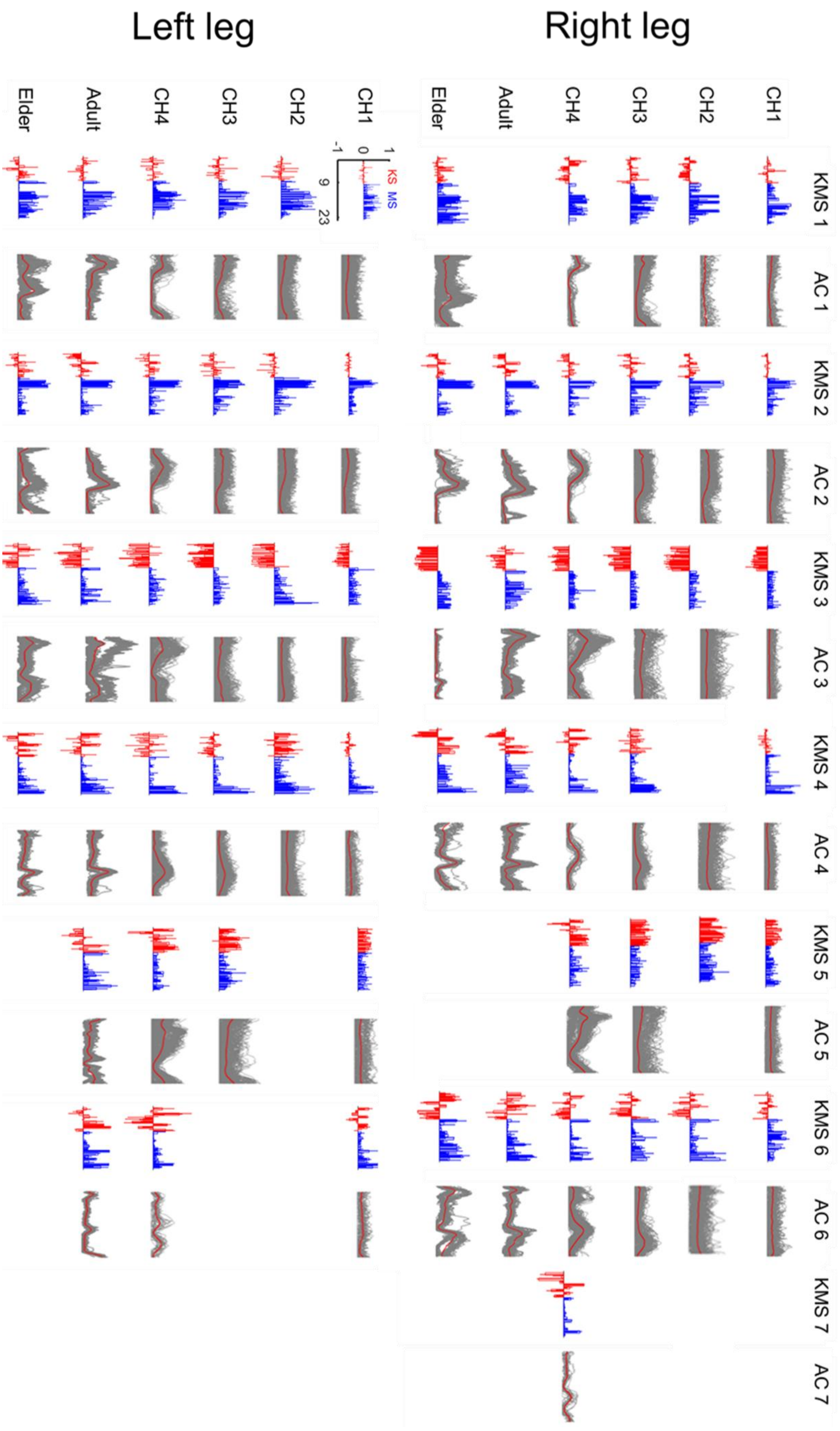

Left leg
Right leg
CH1
CH2
CH3
CH4
Adult
Elder
KMS 1
AC 1
KMS 2
AC 2
KMS 3
AC 3
KMS 4
AC 4
KMS 5
AC 5
KMS 6
AC 6
KMS 7
AC 7
KS
MS
1
0
-1
9
23

Fig. S8. KMS Clusters and Associated ACs Across the Lifespan. *K*-means clustering was performed on all KMSs from the left or right leg of subjects at each of six developmental stages (denoted on the left). The optimal number of KMS clusters was determined by the highest silhouette value (see Methods: *K*-means Clustering). Within each cluster, individual KMS members are shown as bars (KS: red; MS: blue). The ACs associated with each KMS cluster are plotted in gray on the right, with the cluster mean shown in red. The arrangement of KMS clusters and their associated ACs is indicated at the top. Within KMS clusters, MS profiles remained relatively consistent, whereas KS profiles exhibited greater variability across developmental stages, despite minor fluctuations in the number of KMS clusters. For the ACs, population-level variability led to mean AC profiles lacking distinct peaks at CH1 and CH2. In later stages, ACs became more consistent throughout the gait cycle, resulting in mean temporal profiles with clear, consistent peaks. Across these later stages, the averaged ACs of matched KMS clusters also displayed similar temporal profiles, reflecting robust and stable temporal modulation of KMS clusters with development.

Table S1. Regression of Muscle-Tendon Parameters on the Infant's Age

Muscles \ Parameters	ShapeFactor		OptimalFibreLength		TendonSlackLength		StrengthCoefficient	
	Formula	p value	Formula	p value	Formula	p value	Formula	p value
**TA (R)**	y=−0.15x−1.48	0.89	y=−0.018x+0.17	0.003	y=0.04x+0.21	0.007	y=−0.07x+2.66	0.94
**TA (L)**	y=0.33x−2.63	0.73	y=−0.01x+0.15	0.15	y=0.05x+0.17	0.0008	y=0.77x+0.36	0.39
**MG (R)**	y=−1.33x+2.47	0.25	y=−0.003x+0.08	0.25	y=−0.003x+0.35	0.55	y=0.5	0.20
**MG (L)**	y=−3.44x+8.23	0.0005	y=−0.002x+0.08	0.50	y=−0.009x+0.37	0.52	y=0.5	0.66
**LG (R)**	y=1.0x−4.5	0.36	y=−0.003x+0.09	0.27	y=−0.002x+0.35	0.63	y=0.5	0.20
**LG (L)**	y=−1.52x+2.32	0.17	y=−0.003x+0.09	0.27	y=−0.0017x+0.35	0.74	y=0.5	0.66
**Sol (R)**	y=1.15x−3.6	0.17	y=−0.006x+0.15	0.22	y=−0.004x+0.3	0.63	y=0.2x+0.014	0.18
**Sol (L)**	y=−0.54x+1	0.49	y=−0.005x+0.14	0.24	y=−0.0018x+0.29	0.69	y=0.24x−0.12	0.06
**VL (R)**	y=−0.04x+1.71	0.98	y=−0.02x+0.13	0.07	y=−0.017x+0.13	0.10	y=−0.06x+0.94	0.89
**VL (L)**	y=−0.53x−1.14	0.53	y=−0.02x+0.14	0.005	y=0.012x+0.15	0.06	y=−0.13x+1.1	0.66
**VM (R)**	y=−0.71x+0.87	0.53	y=−0.03x+0.17	0.001	y=0.013x+0.1	0.25	y=−0.057x+0.94	0.89
**VM (L)**	y=2.52x−7.13	0.06	y=−0.013x+0.13	0.14	y=0.02x+0.08	0.08	y=−0.13x+1.1	0.66
**RF (R)**	y=−2.32x+5.54	0.012	y=−0.016x+0.18	0.03	y=0.07x+0.1	0.0002	y=0.11x+0.23	0.09
**RF (L)**	y=−3.47x+8.59	0.0002	y=−0.006x+0.15	0.30	y=0.12x−0.02	4.3e−06	y=0.073x+0.32	0.08
**Ham (R)**	y=0.69x−3.16	0.52	y=−0.004x+0.17	0.17	y=−0.05x+0.24	2.11e−06	y=3.73x−7.52	0.0005
**Ham (L)**	y=0.71x−3.44	0.51	y=−0.004x+0.17	0.17	y=−0.05x+0.24	2.02e−06	y=2.79x−4.88	0.015
**AL (R)**	y=0.34x−0.98	0.29	y=−0.013x+0.13	0.08	y=0.03x+0.05	2.04e−07	y=−0.08x+0.73	0.43
**AL (L)**	y=0.73x−2.19	0.26	y=−0.016x+0.14	0.01	y=0.03x+0.06	1.83e−06	y=−0.16x+0.99	0.68
**TFL (R)**	y=2.16x−8.43	0.06	y=−0.003x+0.33	0.55	y=−0.001x+0.12	0.55	y=−1.11x+5.51	0.32
**TFL (L)**	y=2.09x−8.18	0.07	y=−0.003x+0.33	0.55	y=−0.001x+0.12	0.55	y=−1.24x+5.4	0.32
**GM (R)**	y=1.38x−4.61	0.11	y=−0.004x+0.48	0.55	y=−0.03x+0.2	0.004	y=1.49x−2.35	0.13
**GM (L)**	y=−1.6x+3.48	0.019	y=−0.004x+0.48	0.55	y=−0.03x+0.21	0.003	y=3.32x−6.89	0.0005
**ES (R)**	y=−0.65x+1.62	0.07	y=0.008x+0.06	0.08	y=−0.04x+0.44	0.065	y=0.58x+0.51	0.47
**ES (L)**	y=−1.26x+3.12	0.06	y=0.008x+0.06	0.08	y=−0.04x+0.44	0.065	y=−0.46x+3.16	0.57
**ExtO (R)**	y=1.32x−4.99	0.09	y=−0.0007x+0.19	0.97	y=−0.009x+0.28	0.58	y=−0.42x+3.12	0.56
**ExtO (L)**	y=−0.61x+0.16	0.35	y=−0.02x+0.25	0.11	y=0.003x+0.25	0.79	y=1.13x−0.87	0.12

Supplementary References

1. Fig. S1. Between-Stage Synergy Similarity
2. Fig. S2. Sparseness of MS Across the Lifespan.
3. Fig. S3. Rate of Synergy Change Between Stages.
4. Fig. S4. *K*-means Clustering of KMSs From the Right Leg.
5. Fig. S5. *K*-means Clustering on KSs.
6. Fig. S6. Evaluating the Accuracy of NMS Model.
7. Fig. S7. Convergence of AC Patterns Across Infant Locomotor Development.
8. Fig. S8. KMS Clusters and Associated ACs Across the Lifespan.
9. Table S1. Regression of Muscle-Tendon Parameters on the Infant's Age
10. Data1-BetweenStage Comparison.xlsx
11. Data2-BetweenLimb Comparison.xlsx
12. Data3-Within-cluster Subject Comparison.xlsx
13. Data4-Muscle-tendon Parameters.xlsx
14. Data5-AC comparison.xlsx
