# Supplementary material for: Functional symmetrization of neuromotor modules during locomotor development in human infants
Source: Commun Biol. 2025 Dec 18;8:1782. doi: 10.1038/s42003-025-09198-y (PMC12714737; doi:10.1038/s42003-025-09198-y)
Supplement: Supplementary file 3 — Description of Additional Supplementary Files [file 42003_2025_9198_MOESM3_ESM.pdf]

Description of Additional Supplementary Files

File name: Supplementary Data 1: Between Stage Comparison
Description: The source data behind Fig. 2 in the paper.

File name: Supplementary Data 2: Between Limb Comparison
Description: The source data behind Fig. 3 in the paper.

File name: Supplementary Data 3: Within-cluster Subject Comparison
Description: The source data behind Fig. 4 in the paper.

File name: Supplementary Data 4: Muscle-tendon Parameters
Description: The source data behind Fig. 6 in the paper.

File name: Supplementary Data 5: AC comparison
Description: The source data behind Fig. 8 in the paper.
